# Supplementary material for: Analysis of mitochondrial m1A/G RNA modification reveals links to nuclear genetic variants and associated disease processes
Source: Commun Biol. 2020 Mar 27;3:147. doi: 10.1038/s42003-020-0879-3 (PMC7101319; doi:10.1038/s42003-020-0879-3)
Supplement: Supplementary file 1 — Supplementary Material [file 42003_2020_879_MOESM1_ESM.pdf]

Supplementary Tables

Supplementary Table 1: Description of datasets and tissue types used in study.

Project	Data Sources	Tissue	Pre-QC Sample Size	Post-QC Sample Size
**CARTaGENE Project**	Data were obtained through application to the data access committee at CARTaGENE (www.cartagene.qc.ca).	Whole Blood	1010	799
**NIMH Genomics Resource**	Data were obtained through application to the data access committee at the NIMH Genomics Resource (www.nimhgenetics.org).	Whole Blood	1246	908
**Geuvadis Project**	RNA sequence data were downloaded from the European Nucleotide Archive (ERA169774) and DNA sequence data were downloaded from the 1000 Genomes FTP site (v5a.20130502).	LCL	463	435
**TwinsUK Project**	Data were obtained through application to the data access committee at TwinsUK (www.twinsuk.ac.uk) and were downloaded from the European Genome-Phenome Archive (https://ega-archive.org, EGAS00001000805).	Whole Blood	391	363
		Adipose	685	654
		Skin	672	653
		LCL	765	735
**GTEx Project**	Data were obtained through application to dbGaP (phs000424.v6.p1).	Adipose - Subcutaneous	386	308
		Adipose - Visceral Omentum	235	194
		Adrenal Gland	161	132
		Artery - Aorta	250	217
		Artery - Coronary	141	124
		Artery - Tibial	363	303
		Brain - Amygdala	83	63
		Brain - Anterior cingulate cortex (BA24)	100	80
		Brain - Caudate (basal ganglia)	135	111
		Brain - Cerebellar Hemisphere	120	96
		Brain - Cerebellum	147	109
		Brain - Cortex	133	104
		Brain - Frontal Cortex (BA9)	122	96

		Brain - Hippocampus	104	88
		Brain - Hypothalamus	104	85
		Brain - Nucleus accumbens (basal ganglia)	125	100
		Brain - Putamen (basal ganglia)	105	86
		Brain - Spinal cord (cervical c-1)	76	58
		Brain - Substantia nigra	72	60
		Breast - Mammary Tissue	222	176
		Cells - EBV-transformed lymphocytes	139	125
		Cells - Transformed fibroblasts	305	282
		Colon - Sigmoid	175	145
		Colon - Transverse	211	175
		Esophagus - Gastroesophageal Junction	177	145
		Esophagus - Mucosa	338	277
		Esophagus - Muscularis	291	241
		Heart - Atrial Appendage	219	180
		Heart - Left Ventricle	274	212
		Lung	381	279
		Muscle - Skeletal	478	377
		Nerve - Tibial	335	277
		Pancreas	203	167
		Skin - Not Sun Exposed (Suprapubic)	273	216
		Skin - Sun Exposed (Lower leg)	399	321
		Stomach	210	176
		Testis	209	176
		Thyroid	368	290
		Whole Blood	456	354

Supplementary Table 2: Significant associations between nuclear genetic variation and mitochondrial RNA methylation level for single tissue GWASs.

Tissue	Position	rsID	CHR	BP	A1	BETA	P	SNP Type	Nearby Genes	Mediator Genes
Artery - Aorta	Position 2617	rs10865508	2	29053704	C	0.04966	5.30E-26	Intronic	*SPDYA*	NA
Nerve - Tibial	Averaged tRNA P9	rs11156878	14	35735967	G	0.05689	1.77E-21	Missense	*MRPP3*	NA
Nerve - Tibial	Position 9999	rs1963154	14	35815049	T	0.08234	5.96E-19	Intergenic	*PSMA6, NFKBIA*	NA
Artery - Tibial	Position 2617	rs11685682	2	29056801	A	0.05974	4.92E-17	Intronic	*SPDYA*	NA
Esophagus - Muscularis	Position 2617	rs10198789	2	28968811	C	0.03446	5.12E-17	Intergenic	*LOC100505774, PPP1CB*	NA
Nerve - Tibial	Position 10413	rs8018597	14	35771949	A	0.1037	1.06E-16	Intronic	*PSMA6*	NA
Nerve - Tibial	Position 2617	rs34611659	2	29094722	TA	0.05288	1.55E-15	Intergenic	*TRMT61B, WDR43*	NA
Esophagus - Gastroesophageal Junction	Position 2617	rs4132617	2	29031312	C	0.03988	4.05E-14	Intergenic	*PPP1CB, SPDYA*	NA
Nerve - Tibial	Position 12146	rs7158706	14	35736692	A	0.07413	6.10E-13	Intronic	*MRPP3*	NA
Cells - Transformed Fibroblasts	Position 2617	rs13033423	2	29097013	G	-0.04013	9.25E-13	Intergenic	*TRMT61B, WDR43*	NA
Adipose - Visceral Omentum	Position 2617	rs34611659	2	29094722	TA	0.0387	4.11E-12	Intergenic	*TRMT61B, WDR43*	NA
Skin - Sun Exposed Lower Leg	Position 2617	rs6547873	2	28981983	G	0.04516	4.38E-12	Intronic	*PPP1CB*	NA
Nerve - Tibial	Position 7526	Rs140678103	14	35765525	G	0.09911	6.93E-12	Intronic	*PSMA6*	NA
Nerve - Tibial	Position 8303	Rs8018597	14	35771949	A	0.07774	3.64E-11	Intronic	*PSMA6*	NA
Nerve - Tibial	Position 12274	Rs200541481	14	35761028	TCA	0.04063	4.03E-12	Intronic	*PSMA6*	NA

Supplementary Table 3. Replication analyses to test if significant associations observed in whole blood are present in single dataset tissues. Replication count shows where association is significant in an alternative tissue at P<0.05 (corrected for the number of position-SNP pairs).

Position	rsID	Replication Count	Number of tissues considered	Tissues
Position 585	rs13874	0	14	-
Position 585	rs1475041	1	14	NerveTibial
Position 1610	rs13874	0	34	-
Position 1610	rs11156878	8	34	AdiposeVisceralOmentum, ColonSigmoid, ColonTransverse, EsophagusGastroesophagealJunction, EsophagusMuscularis, HeartLeftVentricle, MuscleSkeletal, NerveTibial
Position 3238	rs13874	0	14	-
Position 3238	rs61988267	7	14	BrainAnteriorcingulatecortexBA24, BrainCaudatebasalganglia, BrainCerebellum, BrainCortex, BrainFrontalCortexBA9, BrainPutamenbasalganglia, NerveTibial
Position 4271	rs61988276	5	35	ArteryCoronary, CellsTransformedfibroblasts, ColonTransverse, HeartAtrialAppendage, NerveTibial
Position 5520	rs11156878	9	35	BrainCaudatebasalganglia, BrainCortex, BrainHippocampus, ColonSigmoid, ColonTransverse, EsophagusGastroesophagealJunction, EsophagusMuscularis, HeartLeftVentricle, NerveTibial
Position 7526	rs1084535	1	31	BrainCerebellarHemisphere
Position 7526	rs140678103	10	31	BrainCaudatebasalganglia, BrainCerebellum, BrainCortex, BrainPutamenbasalganglia, ColonSigmoid, EsophagusGastroesophagealJunction, EsophagusMuscularis, HeartAtrialAppendage, Lung, NerveTibial
Position 8303	rs74422990	13	35	BrainCaudatebasalganglia, BrainCerebellum, BrainCortex, BrainHippocampus, BrainPutamenbasalganglia, ColonSigmoid, ColonTransverse, EsophagusGastroesophagealJunction, EsophagusMucosa, HeartLeftVentricle, Lung, NerveTibial, SkinSunExposedLowerleg

Position 9999	rs11156878	15	27	AdiposeVisceralOmentum, ArteryCoronary, ArteryTibial, BrainAnteriorcingulatecortexBA24, BrainCaudatebasalganglia, BrainCerebellarHemisphere, BrainCerebellum, BrainCortex, BrainHippocampus, BrainPutamenbasalganglia, ColonSigmoid, ColonTransverse, EsophagusMuscularis, MuscleSkeletal, NerveTibial
Position 9999	rs11085147	0	27	-
Position 10413	rs3820190	0	34	-
Position 10413	rs11156878	16	34	AdiposeVisceralOmentum, ArteryCoronary, BrainAnteriorcingulatecortexBA24, BrainCaudatebasalganglia, BrainCerebellum, BrainCortex, BrainHippocampus, BrainPutamenbasalganglia, ColonSigmoid, ColonTransverse, EsophagusMucosa, EsophagusMuscularis, HeartLeftVentricle, MuscleSkeletal, NerveTibial, Stomach
Position 12146	rs11156878	16	31	AdiposeVisceralOmentum, ArteryTibial, BrainAnteriorcingulatecortexBA24, BrainCaudatebasalganglia, BrainCerebellum, BrainCortex, BrainHippocampus, BrainPutamenbasalganglia, BreastMammaryTissue, ColonSigmoid, ColonTransverse, EsophagusMucosa, EsophagusMuscularis, HeartLeftVentricle, MuscleSkeletal, NerveTibial
Position 12146	rs11085147	0	31	-
Position 12274	rs11156878	7	15	BrainAnteriorcingulatecortexBA24, BrainCaudatebasalganglia, BrainCerebellum, BrainCortex, BrainHippocampus, BrainPutamenbasalganglia, NerveTibial
Position 14734	rs11156878	2	13	BrainAnteriorcingulatecortexBA24, BrainCaudatebasalganglia
Position 15896	rs11156878	15	34	AdiposeVisceralOmentum, ArteryCoronary, BrainCaudatebasalganglia, BrainCerebellarHemisphere, BrainCerebellum, BrainCortex, BrainFrontalCortexBA9, BrainHippocampus, BrainHypothalamus, BrainSubstantianigra, ColonSigmoid, EsophagusGastroesophagealJunction, EsophagusMuscularis, HeartAtrialAppendage, NerveTibial

Position 2617	rs11684695	25	35	AdiposeVisceralOmentum, AdrenalGland, ArteryAorta, ArteryCoronary, ArteryTibial, BrainAnteriorcingulatecortexBA24, BrainCaudatebasalganglia, BrainCortex, BrainHippocampus, BrainPutamenbasalganglia, BreastMammaryTissue, CellsTransformedfibroblasts, ColonSigmoid, ColonTransverse, EsophagusGastroesophagealJunction, EsophagusMucosa, EsophagusMuscularis, HeartAtrialAppendage, Lung, MuscleSkeletal, NerveTibial, Pancreas, SkinSunExposedLowerleg, Stomach, Thyroid
Position 2617	rs2627773	0	35	-
Position 2617	rs13874	0	35	-
Position 13710	rs10826790	6	35	ArteryAorta, ColonSigmoid, MuscleSkeletal, NerveTibial, SkinSunExposedLowerleg, Thyroid
Averaged tRNA P9	rs11156878	22	32	AdiposeVisceralOmentum, ArteryCoronary, ArteryTibial, BrainAnteriorcingulatecortexBA24, BrainCaudatebasalganglia, BrainCerebellum, BrainCortex, BrainHippocampus, BrainPutamenbasalganglia, BreastMammaryTissue, ColonSigmoid, ColonTransverse, EsophagusGastroesophagealJunction, EsophagusMucosa, EsophagusMuscularis, HeartLeftVentricle, Lung, MuscleSkeletal, NerveTibial, SkinSunExposedLowerleg, Thyroid

Supplementary Table 4. Overlap between peak nuclear genetic variants (and those in LD) identified in this study with results submitted to the NHGRI-EBI GWAS catalogue.

Dataset	Peak SNP	LD SNP	Peak SNP MAF	LD SNP MAF	R2	DP	PUBMED ID	STUDY	DISEASE/TRAIT	INITIAL SAMPLE SIZE	CHR	BP	P-VALUE
CARTaGENE	rs13874	rs34080181	0.44	0.38	0.46	0.97	30061737	Biobank-driven genomic discovery yields new insight into atrial fibrillation biology.	Atrial fibrillation	60,620 European ancestry cases, 970,216 European ancestry controls	3	66403767	1.00E-10
CARTaGENE	rs1084535	rs34080181	0.41	0.38	0.39	0.95							
TwinsUK	rs10166861	rs4577244	0.43	0.24	0.36	0.92	29058716	Identification of ten variants associated with risk of estrogen-receptor-negative breast cancer.	Breast cancer (estrogen-receptor negative)	14,135 European ancestry cases, 58,126 European ancestry controls	2	28897867	2.00E-09
TwinsUK	rs10865508	rs4577244	0.43	0.24	0.36	0.92							
GTEx	rs34611659	rs4577244	0.42	0.23	0.35	0.92							
GTEx	rs13033423	rs4577244	0.44	0.23	0.22	0.94							
CARTaGENE	rs2627773	rs1975487	0.48	0.48	0.87	0.94	27618452	The genetics of blood pressure regulation and its target organs from association studies in 342,415 individuals.	Diastolic blood pressure	up to 201,529 European ancestry individuals	2	55581918	2.00E-09
CARTaGENE	rs2627773	rs2627761	0.48	0.19	0.24	0.96	29891935	A multiethnic genome-wide association study of primary open-angle glaucoma identifies novel risk loci.	Glaucoma (primary open-angle)	10,523 European ancestry cases, 203,579 European ancestry controls.	2	55705879	6.00E-10
GTEx	rs11685682	rs147972440	0.41	0.13	0.21	1.00	29617998	Genome-Wide Association Analyses Identify New Loci Influencing Intraocular Pressure.	Intraocular pressure	115,486 European ancestry individuals	2	28859270	5.00E-08
GTEx	rs34611659	rs147972440	0.42	0.13	0.21	1.00							

GTEx	rs4132617	rs147972440	0.43	0.13	0.20	1.00							
CARTaGENE	rs1475041	rs8016947	0.18	0.48	0.22	0.94	23143594	Identification of 15 new psoriasis susceptibility loci highlights the role of innate immunity.	Psoriasis	10,588 European ancestry cases, 22,806 European ancestry controls	14	35363460	3.00E-17
CARTaGENE	rs11156878	rs8016947	0.18	0.48	0.21	0.91							
CARTaGENE	rs61988267	rs8016947	0.19	0.48	0.21	0.91							
CARTaGENE	rs74422990	rs8016947	0.18	0.48	0.21	0.91							
TwinsUK	rs200541481	rs8016947	0.15	0.43	0.21	0.94							

Supplementary Table 5: m1A/G sites used in this study that have been detected via other high-throughput approaches based around m1Aseq.

mtDNA Position	Study/Resource		
	Li *et al* (2017)[1]	Safra *et al* (2017)[2]	Clark *et al* (2016)[3]
585	Yes	Yes	Yes
1610	Yes	Yes	Yes
3238	No	No	Yes
4271	No	No	Yes
5520	Yes	Yes	Yes
7526	Yes	Yes	Yes
8303	Yes	Yes	Yes
9999	Yes	Yes	Yes
10413	Yes	Yes	Yes
12146	Yes	No	Yes
12274	Yes	Yes	Yes
14734	No	No	Yes
15896	Yes	Yes	Yes
2617	Yes	Yes	NA
13710	Yes	Yes	NA

Supplementary Figures

Supplementary Figure 1. Boxplots of mitochondrial RNA methylation levels across positions and datasets. WBL:Whole Blood, ASU:Adipose Subcutaneous, SNE:Skin Not sun Exposed, LCL:Lymphoblastoid Cell Line, AVO:Adipose Visceral Omentum, AGL:Adrenal Gland, AAO:Artery Aorta, ACO:Artery Coronary, ATI:Artery Tibial, BAM:Brain Amygdala, BAC:Brain Anterior cingulate cortex, BCB:Brain Caudate basal ganglia, BCH:Brain Cerebellar Hemisphere, BCE:Brain Cerebellum, BCO:Brain Cortex, BFC:Brain Frontal Cortex, BSC:Brain Spinal cord cervical, BSN:Brain Substantia nigra, BHI:Brain Hippocampus, BHY:Brain Hypothalamus, BNA:Brain Nucleus accumbens basal ganglia, BPB:Brain Putamen basal ganglia, BMT:Breast Mammary Tissue, CTF:Cells Transformed Fibroblasts, CSI:Colon Sigmoid, CTR:Colon Transverse, EGJ:Esophagus Gastroesophageal Junction, EMUC:Esophagus Mucosa, EMUS:Esophagus Muscularis, HAA:Heart Atrial Appendage, HLV:Heart Left Ventricle, LUN:Lung, MSK:Muscle Skeletal, NTI:Nerve Tibial, PAN:Pancreas, SSE:Skin Sun Exposed, STO:Stomach, TES:Testis, THY:Thyroid

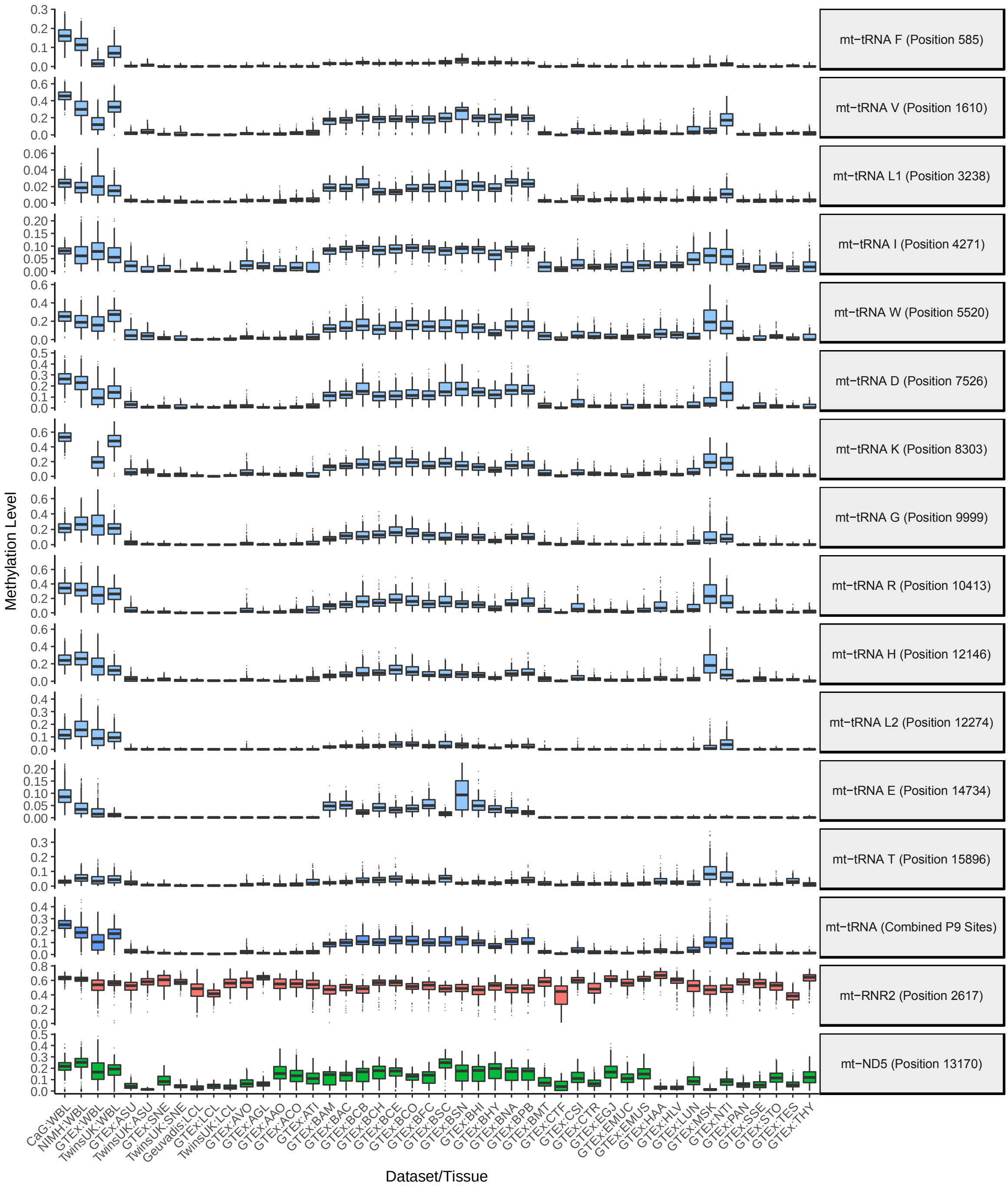

Supplementary Figure 2. RNA sequencing mismatch levels at RNA m1A/G modified sites between samples that have been sequenced two independent times within the CARTaGENE project (N=47). Fifteen sites along the mitochondrial genome were considered per sample (if coverage >20X, see methods for details). Mismatch levels correlate significantly between replicates (R=0.97, P<2.2e-16).

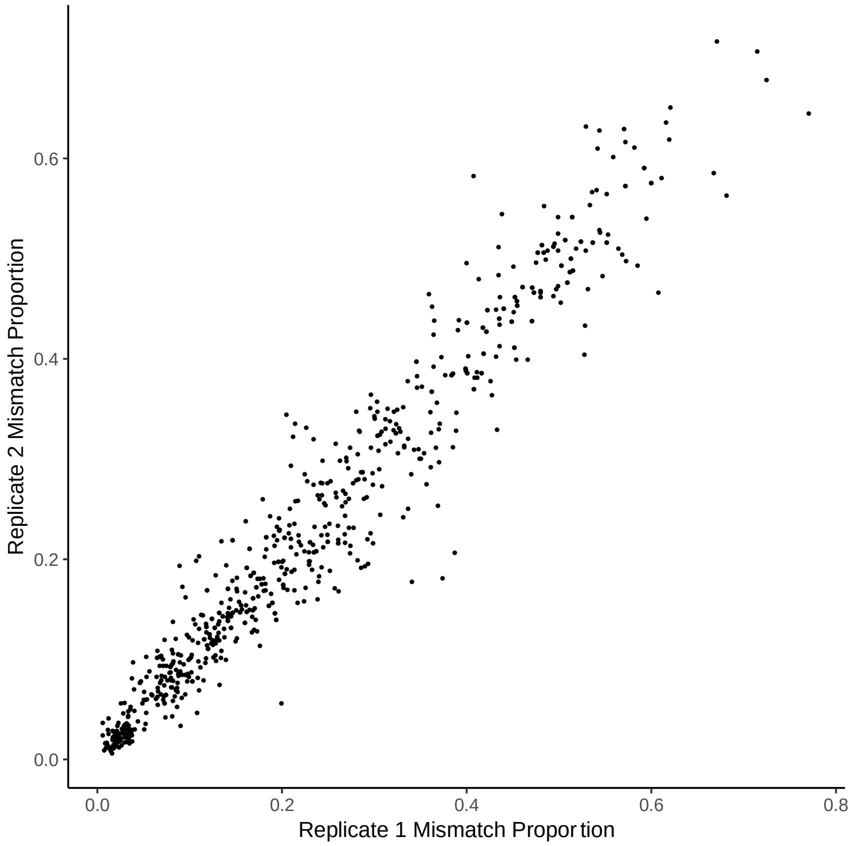

References

1 Li, X. *et al.* Transcriptome-wide mapping reveals reversible and dynamic N(1)-methyladenosine methylome. *Nat Chem Biol* **12**, 311-316, doi:10.1038/nchembio.2040 (2016).

2 Safra, M. *et al.* The m1A landscape on cytosolic and mitochondrial mRNA at single-base resolution. *Nature* **551**, 251-255, doi:10.1038/nature24456 (2017).

3 Clark, W. C., Evans, M. E., Dominissini, D., Zheng, G. & Pan, T. tRNA base methylation identification and quantification via high-throughput sequencing. *RNA* **22**, 1771-1784, doi:10.1261/rna.056531.116 (2016).
