# Supplementary material for: Analysis of mitochondrial m1A/G RNA modification reveals links to nuclear genetic variants and associated disease processes
Source: Commun Biol. 2020 Mar 27;3:147. doi: 10.1038/s42003-020-0879-3 (PMC7101319; doi:10.1038/s42003-020-0879-3)
Supplement: Supplementary file 3 — Description of Additional Supplementary Files [file 42003_2020_879_MOESM3_ESM.pdf]

Description of Additional Supplementary Files

File Name: **Supplementary Data 1**
Description: Raw data used to generate Figures 1, 2 and 3
