# Supplementary material for: Analysis of mitochondrial m1A/G RNA modification reveals links to nuclear genetic variants and associated disease processes
Source: Commun Biol. 2020 Mar 27;3:147. doi: 10.1038/s42003-020-0879-3 (PMC7101319; doi:10.1038/s42003-020-0879-3)
Supplement: Supplementary file 4 — Reporting Summary [file 42003_2020_879_MOESM4_ESM.pdf]

nature research

Corresponding author(s): Alan Hodgkinson

Last updated by author(s): Mar 2, 2020

Reporting Summary

Nature Research wishes to improve the reproducibility of the work that we publish. This form provides structure for consistency and transparency in reporting. For further information on Nature Research policies, see Authors & Referees and the Editorial Policy Checklist.

Statistics

For all statistical analyses, confirm that the following items are present in the figure legend, table legend, main text, or Methods section.

n/a	Confirmed	
☐	☒	The exact sample size (*n*) for each experimental group/condition, given as a discrete number and unit of measurement
☐	☒	A statement on whether measurements were taken from distinct samples or whether the same sample was measured repeatedly
☐	☒	The statistical test(s) used AND whether they are one- or two-sided<br>*Only common tests should be described solely by name; describe more complex techniques in the Methods section.*
☐	☒	A description of all covariates tested
☐	☒	A description of any assumptions or corrections, such as tests of normality and adjustment for multiple comparisons
☐	☒	A full description of the statistical parameters including central tendency (e.g. means) or other basic estimates (e.g. regression coefficient) AND variation (e.g. standard deviation) or associated estimates of uncertainty (e.g. confidence intervals)
☐	☒	For null hypothesis testing, the test statistic (e.g. *F*, *t*, *r*) with confidence intervals, effect sizes, degrees of freedom and *P* value noted<br>*Give P values as exact values whenever suitable.*
☒	☐	For Bayesian analysis, information on the choice of priors and Markov chain Monte Carlo settings
☒	☐	For hierarchical and complex designs, identification of the appropriate level for tests and full reporting of outcomes
☐	☒	Estimates of effect sizes (e.g. Cohen's *d*, Pearson's *r*), indicating how they were calculated

Our web collection on statistics for biologists contains articles on many of the points above.

Software and code

Policy information about availability of computer code

Data collection	No software was used for data collection
Data analysis	For RNAseq quality control: FastQC [v0.11.3]<br>For trimming RNAseq reads and mapping: TrimGalore [v0.4.0], PRINSEQ-lite [v 0.20.4], STAR [2.5.2a_modified]<br>For RNAseq read processing: SAMtools [v1.4.1]<br>For expression quantification: HTseq [v0.6.0]<br>For Genotype QC and processing: QTLtools [v1.0], PLINK [v1.90b3.44] , SHAPEIT [v2.r837], IMPUTE2 [v2.3.2], GTOOL [v0.7.5]<br>For QTL mapping: PLINK [v1.9], GEMMA [v0.96], PEER [v1.0]

For manuscripts utilizing custom algorithms or software that are central to the research but not yet described in published literature, software must be made available to editors/reviewers. We strongly encourage code deposition in a community repository (e.g. GitHub). See the Nature Research guidelines for submitting code & software for further information.

Data

Policy information about availability of data

All manuscripts must include a data availability statement. This statement should provide the following information, where applicable:

- Accession codes, unique identifiers, or web links for publicly available datasets
- A list of figures that have associated raw data
- A description of any restrictions on data availability

RNA sequence and genotype data were obtained from five independent, publicly available projects, including:

CARTaGENE: A population-based cohort comprised of people aged between 40-69, from Quebec, Canada. Whole blood samples were collected for RNA sequencing and genotyping. Data were obtained through application to the data access committee (instructions are available at www.cartagene.qc.ca).

NIMH (National Institute of Mental Health) Genomics Resource: Whole blood samples were collected for RNA sequencing and genotyping from the Depression Genes and Networks study. Data were obtained after application to the data access committee (through www.nimhgenetics.org)

Geuvadis Project: LCL samples from the 1000 Genomes cohort were RNA sequenced and raw data were obtained from the European Nucleotide Archive under submission number ERA169774. Mapped DNA sequence data from phase 1 of the project were downloaded from the 1000 Genomes FTP site (v5a.20130502).

TwinsUK Project: Female monozygotic twin pairs, dizygotic twin pairs and singletons, aged between 38-85 were recruited for RNA sequencing and genotyping. Biopsies from subcutaneous adipose tissue and skin were collected, as well as peripheral blood samples for additional generation of lymphoblastoid cell lines (LCLs). Data were obtained through application to the TwinsUK data access committee and then downloaded from the European Genome-Phenome archive (https://ega-archive.org) through study ID EGAS00001000805.

GTEx (Genotype-Tissue Expression) Project59: Multiple tissue samples were collected from deceased individuals for RNA sequence analysis and dense genotyping. Data were obtained by application to dbGaP through accession number phs000424.v6.p1.

Field-specific reporting

Please select the one below that is the best fit for your research. If you are not sure, read the appropriate sections before making your selection.

☒ Life sciences ☐ Behavioural & social sciences ☐ Ecological, evolutionary & environmental sciences

For a reference copy of the document with all sections, see nature.com/documents/nr-reporting-summary-flat.pdf

Life sciences study design

All studies must disclose on these points even when the disclosure is negative.

Sample size	Sample sizes were governed by data availability - we obtained data from all known population based RNA sequencing studies of 'healthy' individuals with associated genotyping data at the time of analysis.
Data exclusions	Some samples were excluding on a quality control basis:<br><br>For genotyping: Duplicate samples, genetic PC outliers, samples with unexpected relatedness and samples with outlying heterozygosity rates were removed, in addition to samples with discrepant reported and genotypic sex information, or ambiguous X chromosome homozygosity estimates. Samples with > 5% missing genotype data were also excluded.<br><br>For RNAseq data: Samples were excluded for having fewer than 5,000,000 remaining reads, fewer than 10,000 mitochondrial reads, rRNA content greater than 30%, RNAseq mismatch percentage greater than 1%, or intergenic read percentage greater than 30%. We also removed visual outliers after computing principle components from genes with average TPM value > 2.
Replication	For four tissue types (whole blood, LCLs, subcutaneous adipose and non-sun-exposed skin) we had multiple independent datasets for the analysis of observed mitochondrial RNA methylation levels. For QTL mapping we combined these data within a meta-analysis.
Randomization	Samples were split by study and tissue type. For QTL analysis, covariates were used within linear models, including genetic principal components, PEER factors, sex and batch information where available.
Blinding	Blinding is not relevant to the study - samples were grouped by study and tissue type.

Reporting for specific materials, systems and methods

We require information from authors about some types of materials, experimental systems and methods used in many studies. Here, indicate whether each material, system or method listed is relevant to your study. If you are not sure if a list item applies to your research, read the appropriate section before selecting a response.

Materials & experimental systems

n/a	Involved in the study	
☒	☐	Antibodies
☒	☐	Eukaryotic cell lines
☒	☐	Palaeontology
☒	☐	Animals and other organisms
☒	☐	Human research participants
☒	☐	Clinical data

Methods

n/a	Involved in the study	
☒	☐	ChIP-seq
☒	☐	Flow cytometry
☒	☐	MRI-based neuroimaging
